# The gain and loss of chromosomal integron systems in the *Treponema* species

**DOI:** 10.1186/1471-2148-13-16

**Published:** 2013-01-22

**Authors:** Yu-Wei Wu, Thomas G Doak, Yuzhen Ye

**Affiliations:** 1 School of Informatics and Computing, Indiana University, Bloomington, IN, 47408, USA; 2 Department of Biology, Indiana University, Bloomington, IN, 47405, USA

**Keywords:** Chromosomal integron, Treponema species, Integron integrase, *attC* site

## Abstract

**Background:**

Integron systems are now recognized as important agents of bacterial evolution and are prevalent in most environments. One of the human pathogens known to harbor chromosomal integrons, the *Treponema* spirochetes are the only clade among spirochete species found to carry integrons. With the recent release of many new *Treponema* genomes, we were able to study the distribution of chromosomal integrons in this genus.

**Results:**

We find that the *Treponema* spirochetes implicated in human periodontal diseases and those isolated from cow and swine intestines contain chromosomal integrons, but not the *Treponema* species isolated from termite guts. By examining the species tree of selected spirochetes (based on 31 phylogenetic marker genes) and the phylogenetic tree of predicted integron integrases, and assisted by our analysis of predicted integron recombination sites, we found that all integron systems identified in *Treponema* spirochetes are likely to have evolved from a common ancestor—a horizontal gain into the clade. Subsequent to this event, the integron system was lost in the branch leading to the speciation of *T*. *pallidum* and *T*. *phagedenis* (the *Treponema sps*. implicated in sexually transmitted diseases). We also find that the lengths of the integron *attC* sites shortened through *Treponema* speciation, and that the integron gene cassettes of *T*. *denticola* are highly strain specific.

**Conclusions:**

This is the first comprehensive study to characterize the chromosomal integron systems in *Treponema* species. By characterizing integron distribution and cassette contents in the *Treponema sps*., we link the integrons to the speciation of the various species, especially to the pathogens *T*. *pallidum* and *T*. *phagedenis*.

## Background

As important agents of bacterial evolution, integrons are genetic elements that aggregate mobile gene cassettes via site-specific recombination. The functional platform of integron systems consists of a site-specific tyrosine recombinase (*intI*), its primary recombination site (*attI*), and a transcriptional promoter for the cassettes [1]. Integrons are capable of acquiring, rearranging, and expressing genes contained in gene cassettes sampled from a near-infinite environmental bank of cassettes [2]. Each cassette carries one or a small number of genes (some cassettes lack open reading frames) linked to a recombination site termed *attC*. The genes in cassettes are highly diverse and mostly of unknown function [1], and they are usually promoterless and hence rely on the integron’s promoter for transcription. Accordingly, insertions of gene cassettes in the cassette array are highly orientation specific such that integron genes are transcribed from the integron promoter. Studies have revealed that gene cassette composition is extremely dynamic within and between environments [2-5] and even between closely related strains as observed in the *Vibrio cholerae* species [6].

There are two primary types of integrons: mobile integrons and chromosomal integrons [1]. Mobile integrons (including integrons belonging to classes 1, 2 and 3, as defined by their respective *intI* genes) are found commonly on plasmids and are characterized by frequent lateral gene transfers (LGT); most other classes of integrons are nonmobile and so confined to chromosomes and specific bacterial lineages, and are therefore called chromosomal integrons [7]. First discovered in the 1980s [8], mobile integrons contain mostly antibiotic resistance genes [9] and their ability to acquire a number of resistance cassettes leads to most clinical multidrug resistance. By contrast, chromosomal integrons were first found in *Vibrio cholerae* in the 1990s [10], and typically carry more gene cassettes than mobile integrons, of more diverse functions. In some species the chromosomal integrons constitute a significant fraction of the genome (for example, the total length of the gene cassette pools from five *Vibrio* chromosomal integrons is equivalent to a small genome) [1,2].

Chromosomal integrons have been found in a wide range of bacterial species and environmental samples. An analysis surveying 603 sequenced bacterial genomes revealed that 9% carried integrons [7]. Phylogenetic analysis of the integron integrase (*IntI*) suggests that integrons are ancient structures that have contributed to the evolution of bacterial genomes for hundreds of millions of years, primarily by vertical inheritance [11,12]. Horizontal transfer of integrons has also been proposed, as discrepancies are found between the 16S rRNA gene tree and the integron integrase tree for the species *Vibrio fischeri*[13], *Shewanella denitrificans*, *Nitrosococcus mobilis*, and the *Xanthomonas* lineage [7]. At a larger scale, two major clades of integrase were identified, and found to be associated with different environments (soil or ocean), consistent with their exchange among bacterial species occupying common environments [7,11].

*Treponema* species belong to the spirochete family and many are involved in human diseases: *T*. *pallidum* is the cause of syphilis and yaws [14,15]; and the oral pathogen *T*. *denticola* is associated with periodontal diseases [16,17]. Different from other chromosomal integron-containing species (*Vibrio cholerae*, *Vibrio vulnificus and Vibrio parahaemolyticus*) that cause serious infectious diseases, *Treponema* species are widely found in healthy human populations [5]. By building and examining the phylogenetic distribution of chromosomal integron-containing species among a wider selection of spirochetes, and a phylogenetic tree of integron integrases (*IntI*) from genomes that contain chromosomal integrons—assisted by an analysis of *attC* sites—we hypothesize that the chromosomal integron system has undergone at least one gain and one loss in the evolutionary history of the *Treponema* species. The gain happened after the speciation leading to the two termite-gut-associated species, *Treponema azotonutricium* and *Treponema primitia*[18]. The persistent existence of the chromosomal integrons in the human-associated *Treponema* species, especially in those associated with oral sites, indicate that these species may gain evolutionary advantages by having integron gene cassettes. The loss happened in the common ancestor of *T*. *pallidum* and *T*. *phagedenis*: the integron system has been entirely deleted in *T*. *pallidum* (which has one of the smallest known bacterial genomes), while in the *T*. *phagedenis* genome several *attC* sites are present, but the *intI* gene seems to have been lost.

## Methods

### Building evolutionary tree

In order to understand the evolutionary history of the *Treponema* species, we collected the 31 marker genes described in [19] for the *Treponema* species, as well as for species serving as out-groups. The accession numbers and websites for downloading the draft genomes are listed in Additional file 1: Table S1. The 31 reference genes were extracted from the complete genomes according to their annotations. For unannotated draft genomes, a similarity-based search method (BLAST) was employed to find the genes in the contigs. We translated the genes into amino acid sequences using the standard codon table and aligned them using MUSCLE [20]—separately for each gene. The aligned amino acid sequences were then concatenated and imported into MEGA5 [21] for tree construction, with the model set to JTT (Jones-Taylor-Thornton model, the default amino acid model for MEGA5). The bootstrap number was 500.

### Detecting integrons

We looked for integron systems by first detecting the presence of the integron *intI*. To find *intI* genes, we used genome annotations as well as performing similarity searches using the *intI* gene sequence from *T*. *denticola* ATCC 35405 as reference. After collecting *intI* gene sequences we built a tree for *intI* from all species that contain integrons, to ask how the integron system has evolved, using the same procedure as for the species tree described above.

Besides looking for *intI*, we also identified *attC* sites in the genomes, using both similarity-search-based and *ab initio* approaches. While *attI* sites are one of the core components of integron systems, they are less conserved than *attC* sites [1], and there is only one *attI* site in each integron. Thus, we focused on the analysis of *attC* recombination sites, taking advantage of their conservation and the fact that there are often multiple *attC* sites in an integron. First, we used eight recombination site sequences that represent all *attC* sites in the *T*. *denticola* ATCC 35405 integron [5] and performed similarity searches with all eight sequences, since not all recombination sites are the same (all *attC* sites in T. *denticola* ATCC 35405 genome can be aligned to at least one of the representative sequences with > 85% sequence identity; the sequences of these eight *attC* sites are shown in Additional file 1: Table S2). Note that these eight representative *attC* sequences have been used to effectively recover *Treponema* integron gene cassettes from human metagenomic samples [5].

Second, we developed an *ab initio* approach to identifying chromosomal integrons, by searching for arrays of potential *attC* sites. Existing computational methods—including the INTEGRALL database [22] and a context-driven approach using a computational grammar [23]—focus on the identification of mobile integrons, especially those of class I, the most widespread and clinically important type. Our method targets chromosomal integrons, utilizing the unique features of the *attC* sites’ secondary structure: a single-stranded *attC* sequence forms a structure with two stems, an R box and a L box (with one protruding G), connected by a loop of varying length [1]. Our method first scans an input genome for regions that are capable of forming the typical *attC* structure, and then uses these regions’ sequences to search for additional potential *attC* sites (which may be degenerate and thus unable to form the typical *attC* structure), and finally we keep only the candidates that form clusters (with at least two *attC* sites within a 10 K region), to reduce false positives.

### Extracting integron gene cassettes

Except for the annotated *T*. *denticola* ATCC 35405 genomes, for which the gene cassettes are described [16], the integron gene cassettes were extracted from the contigs of the draft genomes by identifying the *attC* recombination sites and extracting genes bounded by these sites. First, the *attC* recombination sites were detected using similarity searches with the eight representative *attC* sequences, with identity threshold set to 70% and coverage set to 50%. We then predicted the cassette genes using FragGeneScan [24], and extracted genes that were exactly bounded by two *attC* sites (overlap between *attC* sites and predicted genes was not allowed). We also set the maximum number of genes between any two *attC* sites to three, which is the maximum number of genes so far reported between any two integron recombination sites [16]. Note that this approach has been applied to human metagenome samples and successfully recovered integron gene cassettes from the metagenomes [5], demonstrating that it is able to effectively collect genes from integron gene cassettes for both complete and fragmented genomic sequences.

## Results and Discussion

### Integrons arrived in the *Treponema* species at least once in their evolutionary history

**Figure 1:**
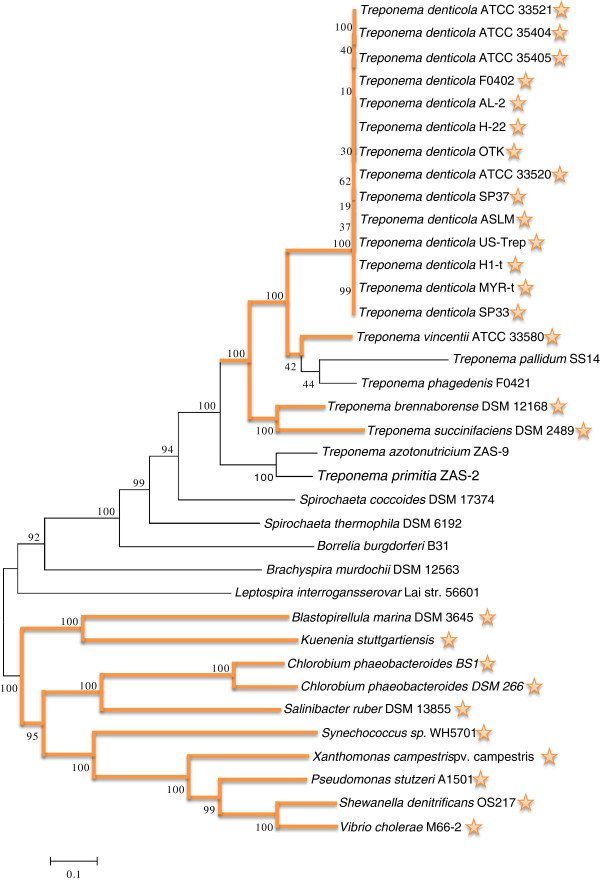
**Species tree based on 31 marker genes.** Stars behind species’ names indicate that the species carry integrons. Orange lines indicate possible integron evolutionary paths during the divergence of the species.

We constructed the phylogenetic tree of the *Treponema* species along with several other spirochete species, as well as several other integron-containing species, as out-groups, as shown in Figure 1. The evolutionary tree suggests that the *Treponema* species appeared after other selected spirochetes, including *Leptospira interrogans*, *Brachyspira murdochii*, *Borrelia burgdorferi*, *Spirochaeta thermophile*, and *Spirochaeta coccoides*. Within the *Treponema* clade, *T*. *azotonutricium* and *T*. *primitia* (both termite associated) are the two earliest branching species among sequenced *Treponema* species.

Integron systems were detected by looking for *intI* genes in sequenced genomes or contigs (see Additional file 1: Figure S1 for a multiple alignment of predicted integron integrases). Figure 1 shows the phylogenetic tree of the species, with integron-containing species marked with stars, and with paths leading to these species highlighted. From this figure one can clearly see that there is a large gap (more than est. 0.4 MYA) between the two subtrees containing integrons (located at the top and bottom part of the figure): integron systems are missing in all earlier branching spirochetes and only appear in some *Treponema* species, including all strains of *T*. *denticola*, *T*. *vincentii*, *T*. *brennaborense*, and *T*. *succinifaciens*. This suggests that the integron system was acquired by the *Treponema* lineage after *T*. *azotonutricium* and *T*. *primitia*—isolated from termite guts—diverged.

**Figure 2:**
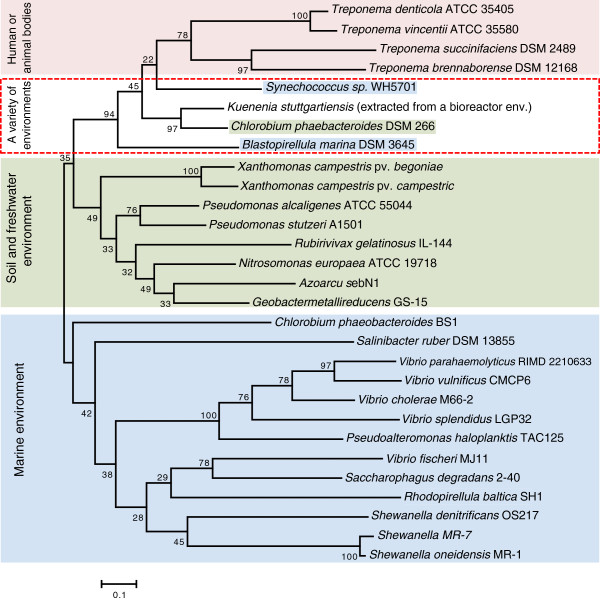
**Evolutionary tree of integron integrase** (***intI***) **genes.** This figure is modeled after Figure two in [11], in which bacteria are classified into two categories: "Marine environment" and "Soil and freshwater environment." We added two more categories, including "Human or animal bodies" to refer to the *Treponema sps*., and “A variety of environments” category, bounded by red dashed box, to represent species with *intI* genes closely-related with the *Treponema* category, but originated from a variety of environments.

We collected *intI* genes from representative species described in [7,11] and built a phylogenetic tree specifically for the *intI* gene, as shown in Figure 2. The *intI* gene sequences of the *Treponema* species form a unique branch in the tree, indicating that the integron system in *Treponema sps*. we identified was obtained via LGT only once, and this branch clusters (boxed in Figure 2) with *intI* genes from *Kuenenia stuttgartiensis*, *Synechococcus* sp., *Chlorobrium phaeobacteroides*, and *Blastopirellula marina*. These species originate from a variety of environments: *Kuennenia stuttgartiensis* was isolated from a bioreactor community; *Chlorobium phaeobacteroides* was isolated from anoxic sulfide-containing water 19.5 m below the surface of the meromictic Lake Blankvann in Norway [25]; and *Synechococcus* sp. and *Blastopirellula marina* were extracted from marine environments; thus the actual transfer path of the integron system to the *Treponema* species is still unclear, as is the division between soil/freshwater and marine integron types suggested in [7,11].

While typical integrons are inverted [7] with the integrase gene transcribed in the opposite direction to that of the cassette genes (the two transcripts diverge from the central *attI* site), the *Treponema* integron loci we have identified share the atypical structure previously characterized in *T*. *denticola*[16] with *intI* transcribed toward the *attI* (and so in the same direction as the cassette genes), adding evidence to the hypothesis of a single LGT of the integron system into the *Treponema* species. We note that there are a few transposases identified in the gene cassettes in the *T*. *succinifaciens* integron (their insertions into the integron could have interrupted the integron structure), but all the genes between the integrase gene (located between 716056 and 717288 bp) and the closest transposase (located between 736651 and 737865 bp)—with the exception of one gene located between 728835 and 728461 bp—are of the same orientation as that of the integrase gene, indicating that the atypical integron structure also applies to *T*. *succinifaciens*.

### Loss of integron systems in the evolutionary history of *Treponema* species

Besides the early gain of integrons by the *Treponema* genus, we also observe the apparent loss of integrons in some *Treponema* species. The most parsimonious explanation is that—following a single gain into the *Treponema* lineage—there was a single loss in the common ancestor of *T*. *pallidum* and *T*. *phagedenis*. To the best of our knowledge, this loss of an integron system from a lineage is unique—the structure of the *Treponema* chromosomal integron and its flanking regions are not associated with mobile elements and do not appear to be mobile [1]. Thus our result provides the first instance of the loss of a chromosomal integron in evolutionary history.

Perhaps the most striking result is observed in the genome of *T*. *phagedenis*. Even though we could not find an *intI* in this species, we did find integron *attC* recombination sites at a chromosomal location syntenic to the integron element in other *Treponema*, and were able to predict integron cassette genes bounded by *attC* sites (see a comparison between the *T*. *phagedenis* genome and the *T*. *denticola* genome in Additional file 1: Figure S2, which highlights the presence of *attC* recombination sites and the absence of *intI* gene in the *T*. *phagedenis* genome). The existence of integron recombination sites and gene cassettes indicates that the integron system did exist in the common ancestor of this species and was lost recently in the evolutionary process. We could not find any traces of the integron structure, including *intI* integrase gene and *attC* recombination sites, in *T*. *pallidum*, suggesting that the integron system has been entirely deleted in this species.

### Differences of *attC* recombination sites between *Treponema* species

**Figure 3:**
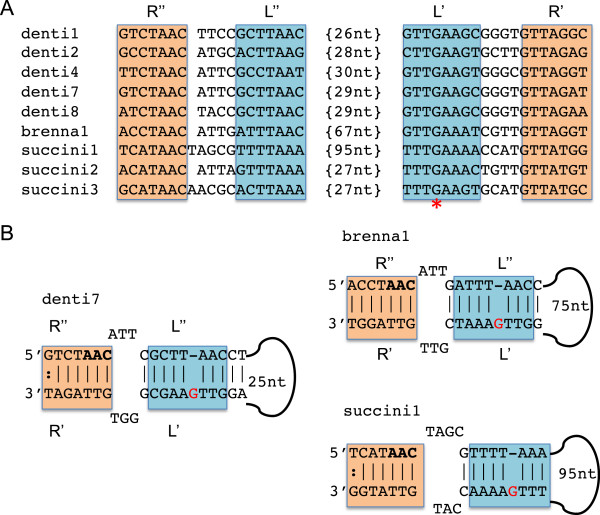
**Analyses of predicted *attC *sites.** (**A**) Multiple alignment of *attC* sites, focusing on the core sites R”, L”, L’, and R’. The protruding G (red asterisk) is highlighted. (**B**) shows the predicted structures of the *attC* sites identified in *T*. *denticola* (denti7), *T*. *phagedenis* (brenna1), and *T*. *succinifaciens* (succini1). We selected the most representative *attC* sites for demonstration purposes: at least 33 *attC* sites identified in *T*. *denticola* share 90% sequence identify over 55 bp with denti7, and in the *T*. *succinifaciens* genome, another five *attC* sites share high sequence similarity (with 92–96% sequence identity) with succini1.

By employing the eight consensus sequences of the *attC* recombination sites to perform similarity searches in the *Treponema* genomes, we found that all *T*. *denticola* strains and *T*. *vincentii* possess these *attC* sites. However, the same method didn’t identify *attC* sites in the *T*. *succinifaciens* and *T*. *brennaborense* genomes. After applying an *ab initio* method to find *attC* sites in the genomes of these two species, we discovered 10 *attC* sites in *T*. *succinifaciens* (from which 3 representative sequences were inferred), and one *attC* site in *T*. *brennaborense*. Figure 3A shows the alignment of predicted *attC* sites; we focused on the core domains of the *attC* sites for the alignment, considering that the sequence conservation in *attC* sites is restricted to two triplets (AAC and GTT) located in the R” and R’ boxes [1]. We only included five *T*. *denticola attC* representative sequences in the alignment, since the other three lack identifiable AAC sites even though they all share sequence similarity with the *attC* sites included in the alignment. Figure 3B shows the predicted structures of these identified *attC* sites: all display the typical stem-loop structure with R and L boxes, suggesting that the *attC* sites found in the *T*. *succinifaciens* and *T*. *brennaborense* genomes are genuine.

Note that we detected only a single *attC* site in the *T*. *brennaborense* genome (between 1344413 and 1344528 bp). However, there is an adjacent *intI* gene (predicted between 1342819 and 1344015 bp; see Additional file 1: Figure S1 for the multiple alignment of the predicted integron integrases, and the conservation of key residues among these sequences). Between the *intI* gene and the *attC* site, we found a small segment (between 1344230 and 1344237 bp) of the same sequence (GTTAGGT) as the R’ binding site in the predicted *attC* site of *T*. *brennaborense*, consistent with this segment being the R binding site of the *attI* site for this integron. All suggest that the integron system in this genome is functional (with integrase gene, *attI* and *attC* sites). Chromosomal integron systems lacking integron gene cassettes are found in other species [1], but the lack of a gene cassette array, which is typical of chromosomal integron structures found in other *Treponema* species, suggests that the integron system in *T*. *brennaborense* needs to be examined in additional isolates.

We found that some of the predicted *attC* sites in *T*. *succinifaciens* and *T*. *brennaborense* are significantly longer than those in *T*. *denticola* and *T*. *vincentii* (in which *attC* sites are 63–68 bps [1]): the lengths of the three *attC* site types in *T*. *succinifaciens* are 63 bps, 68 bps, and 132 bps and the *attC* in *T*. *brennaborense* is 115 bps. Although chromosomal integron arrays—especially those with many gene cassettes—typically contain *attC* sites of similar sizes, arrays have been found that contain *attC* sites of varying lengths [1]. Note that there are more, long *attC* sites (6 out of 10) than short ones in the *T*. *succinifaciens* genome, and these long *attC* sites share high sequence similarity (with 92–96% sequence identity). Since the integron system was inserted into the genome of a common ancestor of the *Treponema* species (as suggested by Figure 1), and *T*. *succinifaciens* and *T*. *brennaborense* appeared earlier than *T*. *denticola* and *T*. *vincentii* (and there are more long *attC* sites in *T*. *succinifaciens*), we hypothesize that the original integron recombination sites were longer (> 100 bps), and were gradually reduced in *T*. *denticola* (63–68 bps).

### Dynamics of integron gene cassettes

**Figure 4:**
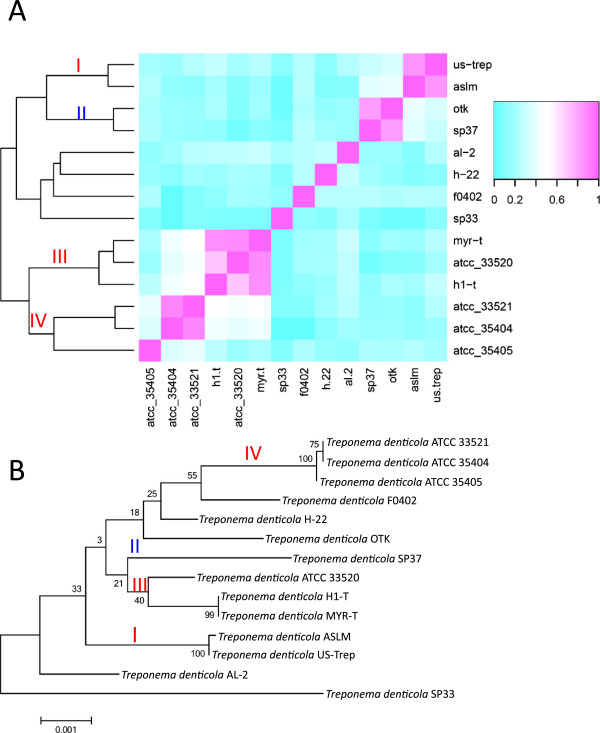
**Grouping of *T. denticola *strains based on shared integron gene cassettes and their phylogenetic relationship.** (**A**) Heat map showing the percentage of shared genes between the 14 *Treponema denticola* strains, with the color spectrum on the right hand side indicating the percentage. The phylogenetic tree shown on the left side of the heat map was generated based on the sharing of the gene cassettes. (**B**) The phylogenetic tree of the 14 *T*. *denticola* strains, built using the 31 marker genes described in Materials and Methods. Roman numerals in (**A**) indicate the groups that share significantly more genes as compared to other strains: red numerals indicate that the groupings are consistent with the phylogenetic relationship in (**B**), and the blue numeral (i.e., group II) highlights an unusual sharing of gene cassettes between not-so-closely-related strains OTK and SP37.

With the availability of the 14 *T*. *denticola* strains, we are able to compare the gene contents of the integron systems between the different strains (Additional file 1: Table S3 lists *attC* recombination sites; Additional file 1: Table S4 and Additional file 1: Table S5 list the details and the total number of the integron cassette genes found in these strains). We calculated the proportion of integron genes shared between any two strains by clustering the genes (amino acid identity threshold is set to 70%) and show the results as a heat map in Figure 4A. Among the 14 strains, a few are similar to each other in gene content. For example, the three strains MYR-t, ATTC 33520, and H1-t (shown as group III in Figure 4) share more than 60% of their genes. Other *T*. *denticola* strains that share large numbers of integron genes include US-Trep and ASLM (group I), and OTK and SP37 (group II). By building a tree based on the 31 marker genes for the 14 strains, as shown in Figure 4B, we found that the sharing of integron genes is largely consistent with the evolutionary relationship among the different *T*. *denticola* strains: more closely related strains tend to share more genes. For example, strains ATCC 33521 and ATCC 35404, which share 89.7% of their integron genes, are almost indistinguishable on the phylogenetic tree. Another example is strains US-TREP and ASLM, which share 84.4% of their integron genes and are closest to each other, as compared to other strains. An exception is that of OTK and SP37, which share 82.6% of their integron genes (highlighted as group II in Figure 4A) even though they are not close to each other on the phylogenetic tree (see Figure 4B). A possible explanation is that these two species are exposed to similar resources for cassette exchange. Further investigation is needed to understand this phenomenon.

Despite the fact that very closely related *T*. *denticola* strains share integron genes, the overall integron-gene-sharing among all the *denticola* strains is not very high (on average two *denticola* strains share 24.79% of their integron genes; also see Figure 4A). Note that Koenig *et al*. reported even less sharing of integron genes (< 10%) among 12 *Vibrio* isolates [26]. We also reported in a previous work that *T*. *denticola* integron gene contents are very different between different metagenomic samples [5]. The dynamics of integron genes in *T*. *denticola* indicate that the integrons of *T*. *denticola* are fully active and are undergoing active insertion and deletion of cassettes.

Note that for the study of gene cassette dynamics in *T*. *denticola* (Figure 4A), we only considered genes present between identified *attC* sites, as *attC* sites are easier to predict than *attI* sites (*attI* sites are less conserved). However, we believe that ignoring the very first gene cassettes between the *attI* site and the adjacent *attC* site would not change our conclusions, considering that *T*. *denticola* isolates contain long arrays of gene cassettes.

## Conclusion

Our combined sequence and phylogenetic analysis of the integron integrases and *attC* recombination sites in *Treponema* genomes suggest that integron systems have been acquired only once, by the common ancestor of *T*. *brennaborense*, *T*. *succinifaciens*, *T*. *vincentii and T*. *denticola*, as we could not find traces of integrons in earlier branching *Treponema* species (*T*. *azotonutriciums* and *T*. *primitia*, which are associated with termites) or other spirochetes. We cannot exclude the possibility that the integrons have been gained independently in these species; however a single insertion event is more likely than several distinct events, as the chromosomal integrons are not associated with mobile elements and so cannot move freely, and the *Treponema intI* genes form a common clade (Figure 2).

We also found evidence for the deletion of integrons in one subclade. The absence of a complete integron structure in *T*. *pallidum* and *T*. *phagedenis* can be explained by a single deletion event in the common ancestor of these two species. Moreover, we found remnants of the integron structure, including *attC* recombination sites and gene cassettes, in the *T*. *phagedenis* genome, but not in *T*. *pallidum*. Without the *intI* gene this integron structure is now static and cannot facilitate adaptation to new conditions by acquiring new gene cassettes, shuffling of existing gene cassettes, or deletion of cassettes. We could not find traces of an integron structure in *T*. *pallidum*, which could be the result of complete degradation of its integron structure.

Our results demonstrate both gain and loss of integron systems in the *Treponema* species. Even though the horizontal acquisition of integron structures has been suggested by [7,13], to the best of our knowledge no literature has provided molecular evidence of integron loss—integrons have been found to be evolutionarily stable. The absence of the *intI*—along with the integron remnants in *T*. *phagedenis*—serves as the first instance of an integron deletion event. Since these chromosomal integrons are not associated with mobile elements, the mechanism of integron insertion/deletion is unknown. With the release of ever more bacterial genomes, we may be able to identify more indel events of integron systems and infer the reasons for these events in the foreseeable future.

## Competing interests

There are no competing interests.

## Authors’ contributions

YW conceived of the study, carried out the analysis and drafted the manuscript. TD participated in the analysis and helped to draft the manuscript. YY conceived of the study, and participated in its design and coordination and helped to draft the manuscript. All authors read and approved the final manuscript.

## Supplementary Material

Additional file 1 **Supplementary materials.** This document contains Supplementary Figures S1–S2, and Supplementary Tables S1–S5. Click here for file
