# Supplementary material for: The gain and loss of chromosomal integron systems in the Treponema species
Source: BMC Evol Biol. 2013 Jan 22;13:16. doi: 10.1186/1471-2148-13-16 (PMC3607928; doi:10.1186/1471-2148-13-16)
Supplement: Additional file 1 — Supplementary materials. This document contains Supplementary Figures S1–S2, and Supplementary Tables S1–S5. [file 1471-2148-13-16-S1.pdf]

This document contains Supplementary Figs 1–2, and Supplementary Tables 1–5.

The Gain and Loss of Chromosomal Integron Systems in the *Treponema* Species

Yu-Wei Wu, Thomas G. Doak, and Yuzhen Ye

146 161

T. succinifaciens DSM 2489	NVVHAKKKTRIPVVFSRQEVSKIIGNL-TGTKKLIAKLLYGTGLRLNEALSLRILDIDFD
T. denticola ATCC 35405	NIVRAKKPKKLPAIMTREETAKIFSLLPENDYGLLIRLLYGTGMRLMEALRLRIQDIDFG
T. vincentii ATCC 35580	NIIRAKKSKKLPAVLSREETAKIFSLLPENDYGLFIRLLYGTGMRLMESLRLRVQDIDFD
T. brennaborense DSM 12168	SVIRAKKTVHIPVVFSKDEVRAIFDQL-SDEKLLIAQLLYGTGMRLSECLCLRIQDIDFA
V. cholerae M66-2	RFQRSQLERKLPVVLTRDEIRRLLEIV-DPKHQLPIKLLYGSGLRLMECMRLRVQDIDFD
V. parahaemolyticus RIMD 2210633	RFQKSLTEKKLPVVLTRDEVRRFVQHI-DPRYKLHIQLLYGSGLRIMECLRLRIQDIDYD
V. fischeri MJ11	EFKRSKKATKLPTVFSHSEAISVINEL-NHPTKLIAQLMYGSGLRINEALRLRVDDIDLI
V. vulnificus CMCP6	KFQKSLLDRKLPVVLTKPELRAFIQHI-DPRYKLHIMLLYGSGLRVMECVRLRIQDVDYH
V. splendidus LGP32	QFRRSDKSRKSPTVMTPEEIGRFFNHC-SPNFKLPYQLMYGSGLRLMECLRLRMQDIDYD
P. haloplanktis TAC125	GFIKSKRATKLPVVLTQQEINNFFKVC-SAKHYLPCGLLYGSGMRLMEVLRLRIHDIDFD
P. alcaligenes ATCC 55044	GVIRARRPQRLPVVLTREEVASVLAQL-DGTLWLIASLLYGSGMRLMEVLRLRVKDVEFA
P. stutzeri A1501	DVVRAKKPQRLPVVLTVEEVRSALAQL-ESEPWLVCSLLYGTGMRLMEALRLRVKDVDFS
S. denitrificans OS217	QFPYAKAPKRIPQVLSNEEAKLIISNL-SGYHYLIGAILFGSGLRLKEALKLRVKDIDLT
Shewanella MR-7	GFCYATKQRHLPTVLSPSEISLILNEL-DGRDKLIIELLYGSGLRVSECLRLRIQDIDIE
S. oneidensis MR-1	GFCYATKQRYLPTVLSPSEISLILNEL-DGRDRLIIELLYGSGLRVSECLRLRIQDIDIE
S. degradans 2-40	AYTPSSKHKKLPTVFSHDEAMRVISHL-QGVHQVLVMLMYGSGLRVMEAVRLRVQDVDFA
X. campestris pv. *begoniae*	NLVRAKRPRRIPAVLWREEVARLLAVL-EGPCWLMASLPYGSGMRMMECLRLRIMDVDAA
X. campestris pv. *campestris*	NLVRAKRPRRIPVVLSVEEVTRLLTML-EGACRLMAGLLYGSGMRLLECLRLRIKDVDMV
N. europaea ATCC 19718	KVVRAKQPQRLPVVLTRTEVQAILVRM-SGTYGLMANLLYGTGMRLMECVRLRVKDVDFE
Azoarcus ebN1	GITRARESKRLPVVMTQRETQALLRHV-HGTSGTIIKLLYGTGMRLLEGLRLRVKDIDLE
G. metallireducens GS-15	DVEQAKKPQRLPVVLTVSEVQALLSRL-TGTHALMGRLMYGTGMRLMETVRLRVKDVDFE
R. gelatinosus IL144	EIGRPVPKRRLPVVLSPAEVAAVLDRL-DGTHRLLGELLYGTGLRITEALQLRVKDIDFA
R. baltica SH1	EAIRANKDSMAPTVMSPEEVGQVFEGL-EGVYLVIAKLLYGCGMRISETHRLRVKDIDFA
K. stuttgartiensis	KVIRARKPTRLPVVMTRDEVKAVLSNL-SEDKWLIASLMYGAGLRLMECLRLRVQDIDFS
B. marina DSM 3645	GVVRARRPKRLPVVLTVDEVSRVMVHL-TSDKWLIAMLLYGGGLRLLEALRMRVKDIDFE
C. phaeobacteroides DSM 266	HLIRARKPSHLPVVLTRDEVKALLVQL-AGVKWLMASLMYGAGLRLMECLRLRVQDIDFS
C. phaeobacteroides BS1	ATVRSNRPRKLPVVLSREEVRSILEIM-NEVTWLMASLIYGGGLRIDECLKLRIKDIDFD
Synechococcus sp.	GVVRARQRKRLPVVLTVAEVKAVLSQL-EGVEQLVAQLLYGSGLRLMEALRLRVKDVDCQ
S. ruber DSM 13855	PLDRADRPKRLPTVLSREEVRQLFAALSPGPNRLTAHLLYGSGLRLSEALRLRVKELDVG

168 174 220

T. succinifaciens DSM 2489	RNEIIVRHGKGDKDRHVMIPRTLICELKSHIENLRKIHEEDLKA-GFGSVKLPQALSDKY
T. denticola ATCC 35405	KNEITVHCGKGAKDRKTVLPVSLKFPLQKHMEKVRLIHEADCKE-GFGSVPLPFALAKKY
T. vincentii ATCC 35580	KNEITVHCGKGAKDRKTMLPVSLKFPLQKHLENVRRIHEADCKD-GFGLVPLPSALAKKY
T. brennaborense DSM 12168	HNMIIIRNGKGAKDRRTMLPHILEKKIRAHIVSVKQLHQADIAD-GWGVVTLPFALRKKY
V. cholerae M66-2	YGAIRIWQGKGGKNRTVTLAKELYPHLKEQIALAKRYYDRDLHQKNYGGVWLPTALKEKY
V. parahaemolyticus RIMD 2210633	YGAVRVWQGKGGKNRTVTLAKELHEPLKSQMNLARNYYFKDRHVPGYAGVYISEGLRRKY
V. fischeri MJ11	HKTITIRNGKGNKDRTTVLPDSIIPELKTQLAFVKQQHKMDAIN-GDDEVYLPNAIAKKY
V. vulnificus CMCP6	YGALRVWQGKGGKNRTVTLAKELYPLLKEQEALALRYYQKDRITAGYAGVWLSSSLQRKY
V. splendidus LGP32	YKSVRVWQGKGGKNRIVTIAPELFPAIKLQQQKSAEYYQQDIHDKAFSGVYLPDSLAKKY
P. haloplanktis TAC125	YNCIRIWDGKGGKNRVVTLAVELTPQLRSQIQLVDSYLQLDLKNPLYSGAYMPHLLRKKY
P. alcaligenes ATCC 55044	RLEILIRDGKGQKDRVTMLPRKLAAPLELHLQRVRALHEQDLRE-GYGRANLPHALARKY
P. stutzeri A1501	RHEILIRDGKGMKDRVTMLPRSLVQPLRRHLAVVKAIHDSEREQ-GRGDVWLPFALARKY
S. denitrificans OS217	TKSIFVYRGKGQKDRVCMLPNGLIDVLKSQMKQVKKLHEADLSD-GFGLASLPISLIRKY
Shewanella MR-7	RASLSVRDGKGHKDRQTILSHRCAEKLTTYIEKAIKTQQNDNLR-GIGP-SLPNALERKY
S. oneidensis MR-1	SASLTVRDGKGHKDRQTILSHRCAEKLTTYIEKAIKIQQDDNQQ-GIGP-SLPNALERKY
S. degradans 2-40	NECLMIRESKGLKWRRTLLPKSLIEPLTIYRDIALARHKADLAD-GYGSVYLPNALNVKY
X. campestris pv. *begoniae*	RGEMLVRDGKGGKDRRVPLPHSLRKALQRKRERALLVHVADLAA-GAGRVFLPHALARKY
X. campestris pv. *campestris*	RCEIVVRDGKGGKDRRVPLPRSLRGELMQQRERALLLHAADLAE-GAGQVFLPHALARKY
N. europaea ATCC 19718	RGEILIRDGKGAKDRVTILPESLVSPLQTYLLQRRVLFDDDIRL-GKASVYLPDALERKY
Azoarcus ebN1	RREIIIREGKGNKDRVTMLPASLVDELRDHLQARRVIHDRDLST-GHADVELPHAIERKY
G. metallireducens GS-15	RSEIIVREGKGAKDRITMLPQAVAAPLREHLGRVEALHGADLRA-GFGEVHLPHALGKKY
R. gelatinosus IL144	QRAIVVRAGKGGKDRVVTLPATLEAPLREQLRAVHAVWAEDSAA-GHAGVQLPDPLERKV
R. baltica SH1	NKQIEIRQSKGNKSRLVPMPDDLIEPLRRFVKTRDALHEHDLAN-GTASVYLPYALDRKY
K. stuttgartiensis	RNEILVRDGKGAKDRITMLPELLKKPLQDHLRKVKAIHEKDLAE-GWGRVQMPDALDRKY
B. marina DSM 3645	RSEITIREGKGDKDRVTTMPRAVVHSLQEHLQRVKLVHEQDVAD-GYGRAELPHALARKY
C. phaeobacteroides DSM 266	SNEILVRDGKGAKDRITMLPESLKKPLADHLKQIKSLHDKDLAE-GWGRVLLPGALDRKY
C. phaeobacteroides BS1	RGFLTVRSGKGNKDRQTLFPKNLAEPLRNHLSEIRNIHEEDRRN-GIEGVSMPGALAYKY
Synechococcus sp.	ARQLTVRSGKGDKDRVTMLPTSVVEPVQAYLLAVREIHRADLAA-GWGRVMLPLALGRKY
S. ruber DSM 13855	TSRLHVWDGKGGTDRTTVLPERLHGPLRRHLKTVKAQHEADCAD-GVGGVYLPDAIAEKY

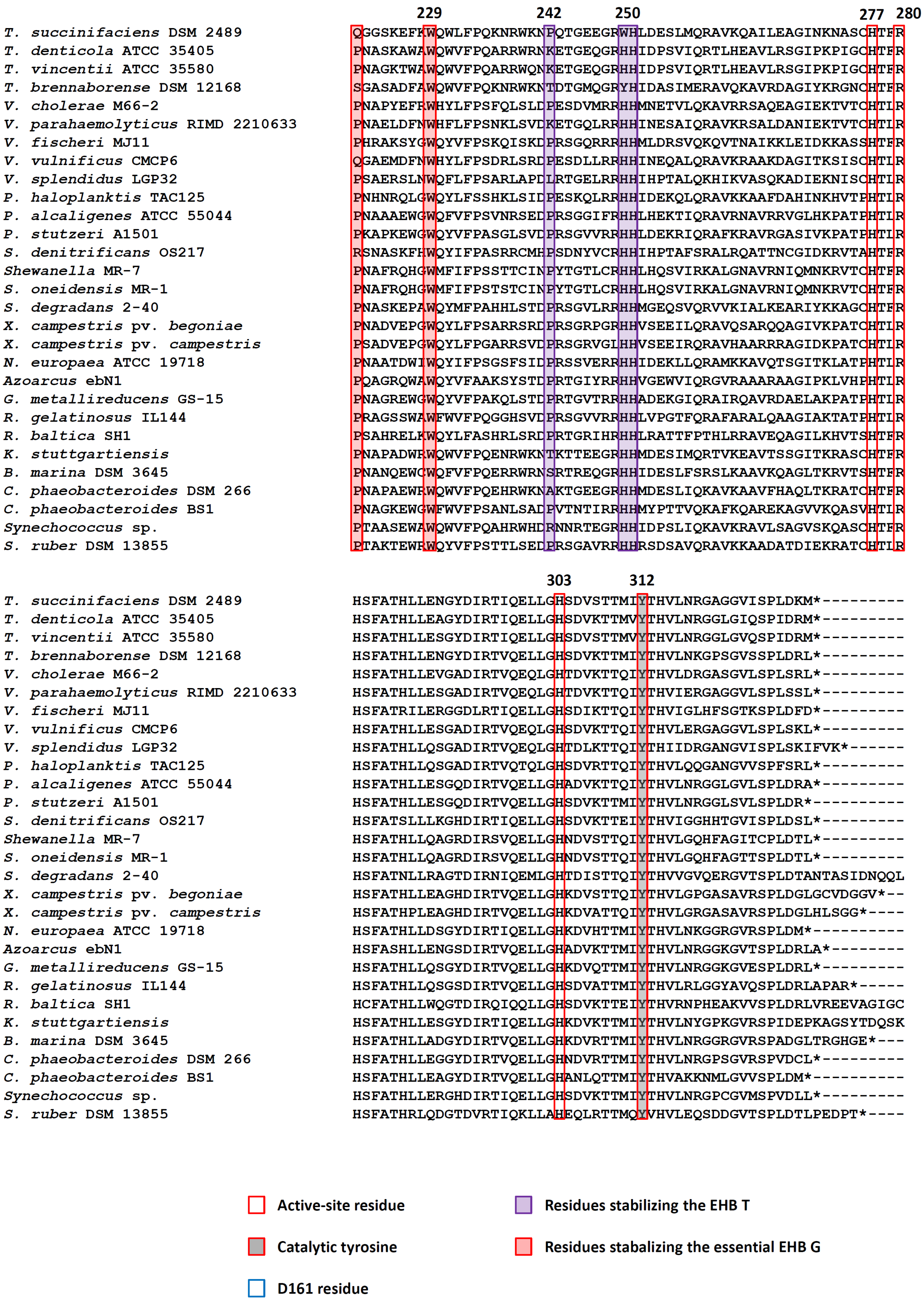

229
242
250
277
280
T. succinifaciens DSM 2489 QGGSKEFKWQWLFPQKNRWKNPQTGEEGRWHLDESLMQRAVKQAILEAGINKNASCHTFR
T. denticola ATCC 35405 PNASKAWAWQWVFPQARRWRNKETGEQGRHHIDPSVIQRTLHEAVLRSGIPKPIGCHTFR
T. vincentii ATCC 35580 PNAGKTWAWQWVFPQARRWQNKETGEQGRHHIDPSVIQRTLHEAVLRSGIPKPIGCHTFR
T. brennaborense DSM 12168 SGASADFAWQWVFPQKNRWKNTDTGMQGRYHIDASIMERAVQKAVRDAGIYKRGNCHTFR
V. cholerae M66-2 PNAPYEFRWHYLFPSFQLSLDPESDVMRRHHMNETVLQKAVRRSAQEAGIEKTVTCHTLR
V. parahaemolyticus RIMD 2210633 PNAELDFNWHFLFPSNKLSVDKETGQLRRHHINESAIQRAVKRSALDANIEKTVTCHTLR
V. fischeri MJ11 PHRAKSYGWQYVFPSKQISKDPRSGQRRRHHMLDRSVQKQVTNAIKKLEIDKKASSHTFR
V. vulnificus CMCP6 QGAEMDFNWHYLFPSDRLSRDPESDLLRRHHINEQALQRAVKRAAKDAGITKSISCHTLR
V. splendidus LGP32 PSAERSLNWQFLFPSARLAPDLRTGELRRHHIHPTALQKHIKVASQKADIEKNISCHTLR
P. haloplanktis TAC125 PNHNRQLGWQYLFSSHKLSIDPESKQLRRHHIDEKQLQRAVKKAAFDAHINKHVTPHTLR
P. alcaligenes ATCC 55044 PNAAAEWGWQFVFPSVNRSEDPRSGGIFRHHLHEKTIQRAVRNAVRRVGLHKPATPHTLR
P. stutzeri A1501 PKAPKEWGWQYVFPASGLSVDPRSGVVRRHHLDEKRIQRAFKRAVRGASIVKPATPHTLR
S. denitrificans OS217 RSNASKFHWQYIFPASRRCMHPSDNYVCRHHIHPTAFSRALRQATTNCGIDKRVTAHTFR
Shewanella MR-7 PNAFRQHGWMFIFPSSTTCINPYTGTLCRHHLHQSVIRKALGNAVRNIQMNKRVTCHTFR
S. oneidensis MR-1 PNAFRQHGWMFIFPSTSTCINPYTGTLCRHHLHQSVIRKALGNAVRNIQMNKRVTCHTFR
S. degradans 2-40 PNASKEPAWQYMFPAHHLSTDPRSGVLRRHHMGEQSVQRVVKIALKEARIYKKAGCHTFR
X. campestris pv. begoniae PNADVEPGWQYLFPSARRSRDPRSGRPGRHHVSEEILQRAVQSARQQAGIVKPATCHTLR
X. campestris pv. campestris PSADVEPGWQYLFPGARRSVDPRSGRVGLHHVSEEIRQRAVHAARRRAGIDKPATCHTLR
N. europaea ATCC 19718 PNAATDWIWQYIFPSGSFSIDPRSSVERRHHIDEKLLQRAMKKAVQTSGITKLATPHTLR
Azoarcus ebN1 PQAGRQWAWQYVFAAKSYSTDPRTGIYRRHHVGEWVIQRGVRAAARAAGIPKLVHPHTLR
G. metallireducens GS-15 PNAGREWGWQYVFPAKQLSTDPRTGVTRRHHADEKGIQRAIRQAVRDAELAKPATPHTLR
R. gelatinosus IL144 PRAGSSWAWFWVFPQGGHSVDPRSGVVRRHHLVPGTFQRAFARALQAAGIAKTATPHTLR
R. baltica SH1 PSAHRELKWQYLFASHRLSRDPRTGRIHRHHLRATTFPTHLRRAVEQAGILKHVTSHTFR
K. stuttgartiensis PNAPADWRWQWVFPQENRWKNTKTTEEGRHHMDESIMQRTVKEAVTSSGITKRASCHTFR
B. marina DSM 3645 PNANQEWCWQFVFPQERRWRNSRTREQGRHHIDESLFSRSLKAAVKQAGLTKRVTSHTFR
C. phaeobacteroides DSM 266 PNAPAEWRWQWVFPQEHRWKNAKTGEEGRHHMDESLIQKAVKAAVFHAQLTKRATCHTFR
C. phaeobacteroides BS1 PNAGKEWGWFWVFPSANLSADPVTNTIRRHHMYPTTVQKAFKQAREKAGVVKQASVHTLR
Synechococcus sp. PTAASEWAWQWVFPQAHRWHDRNNRTEGRHHIDPSLIQKAVKRAVLSAGVSKQASCHTFR
S. ruber DSM 13855 PTAKTEWRWQYVFPSTTLSEDPRSGAVRRHHRSDSAVQRAVKKAADATDIEKRATCHTLR
303
312
T. succinifaciens DSM 2489 HSFATHLLENGYDIRTIQELLGHSDVSTTMIYTHVLNRGAGGVISPLDKM*---------
T. denticola ATCC 35405 HSFATHLLEAGYDIRTIQELLGHSDVKTTMVYTHVLNRGGLGIQSPIDRM*---------
T. vincentii ATCC 35580 HSFATHLLESGYDIRTIQELLGHSDVSTTMVYTHVLNRGGLGVQSPIDRM*---------
T. brennaborense DSM 12168 HSFATHLLENGYDIRTVQELLGHSDVKTTMIYTHVLNKGPSGVSSPLDRL*---------
V. cholerae M66-2 HSFATHLLEVGADIRTVQEQLGHTDVKTTQIYTHVLDRGASGVLSPLSRL*---------
V. parahaemolyticus RIMD 2210633 HSFATHLLESGADIRTVQEQLGHTDVKTTQIYTHVIERGAGGVLSPLSSL*---------
V. fischeri MJ11 HSFATRILERGGDLRTIQELLGHSDIKTTQIYTHVIGLHFSGTKSPLDFD*---------
V. vulnificus CMCP6 HSFATHLLESGADIRTVQEQLGHSDVKTTQIYTHVLERGAGGVLSPLSKL*---------
V. splendidus LGP32 HSFATHLLQSGADIRTVQEQLGHTDLKTTQIYTHIIDRGANGVISPLSKIFVK*------
P. haloplanktis TAC125 HSFATHLLQSGADIRTVQTQLGHSDVRTTQIYTHVLQQGANGVVSPFSRL*---------
P. alcaligenes ATCC 55044 HSFATHLLESGQDIRTVQELLGHADVKTTQIYTHVLNRGGLGVLSPLDRA*---------
P. stutzeri A1501 HSFATHLLESGQDIRTVQELLGHSDVKTTMIYTHVLNRGGLSVLSPLDR*----------
S. denitrificans OS217 HSFATSLLLKGHDIRTIQELLGHSDVKTTEIYTHVIGGHHTGVISPLDSL*---------
Shewanella MR-7 HSFATHLLQAGRDIRSVQELLGHNDVSTTQIYTHVLGQHFAGITCPLDTL*---------
S. oneidensis MR-1 HSFATHLLQAGRDIRSVQELLGHNDVSTTQIYTHVLGQHFAGTTSPLDTL*---------
S. degradans 2-40 HSFATNLLRAGTDIRNIQEMLGHTDISTTQIYTHVVGVQERGVTSPLDTANTASIDNQQL
X. campestris pv. begoniae HSFATHLLEAGHDIRTVQELLGHKDVSTTQIYTHVLGPGASAVRSPLDGLGCVDGGV*--
X. campestris pv. campestris HSFATHPLEAGHDIRTVQELLGHKDVATTQIYTHVLGRGASAVRSPLDGLHLSGG*----
N. europaea ATCC 19718 HSFATHLLDSGYDIRTIQELLGHKDVHTTMIYTHVLNKGGRGVRSPLDM*----------
Azoarcus ebN1 HSFASHLLENGSDIRTVQELLGHADVKTTMIYTHVLNRGGKGVTSPLDRLA*--------
G. metallireducens GS-15 HSFATHLLQSGYDIRTVQELLGHKDVQTTMIYTHVLNRGGKGVESPLDRL*---------
R. gelatinosus IL144 HSFATHLLQSGSDIRTVQELLGHSDVATTMIYTHVLRLGGYAVQSPLDRLAPAR*-----
R. baltica SH1 HCFATHLLWQGTDIRQIQQLLGHSDVKTTEIYTHVRNPHEAKVVSPLDRLVREEVAGIGC
K. stuttgartiensis HSFATHLLESGYDIRTIQELLGHKDVKTTMIYTHVLNYGPKGVRSPIDEPKAGSYTDQSK
B. marina DSM 3645 HSFATHLLADGYDIRTVQELLGHKDVRTTMIYTHVLNRGGRGVRSPADGLTRGHGE*---
C. phaeobacteroides DSM 266 HSFATHLLEGGYDIRTVQELLGHNDVRTTMIYTHVLNRGPSGVRSPVDCL*---------
C. phaeobacteroides BS1 HSFATHLLEAGYDIRTVQELLGHANLQTTMIYTHVAKKNMLGVVSPLDM*----------
Synechococcus sp. HSFATHLLERGHDIRTIQELLGHSDVKTTMIYTHVLNRGPCGVMSPVDLL*---------
S. ruber DSM 13855 HSFATHRLQDGTDVRTIQKLLAHEQLRTTMQYVHVLEQSDDGVTSPLDTLPEDPT*----
Active-site residue
Residues stabilizing the EHB T
Catalytic tyrosine
Residues stabalizing the essential EHB G
D161 residue

Supplementary Figure 1. Multiple alignment of predicted integron integrases from *Treponema* species. The columns corresponding to functionally important sites—including active sites, the catalytic tyrosine, the D161 residue that is important for synapse formation, and residues important for stabilizing the unpaired T and unpaired G—are highlighted in the figure. These sites are marked in [1].

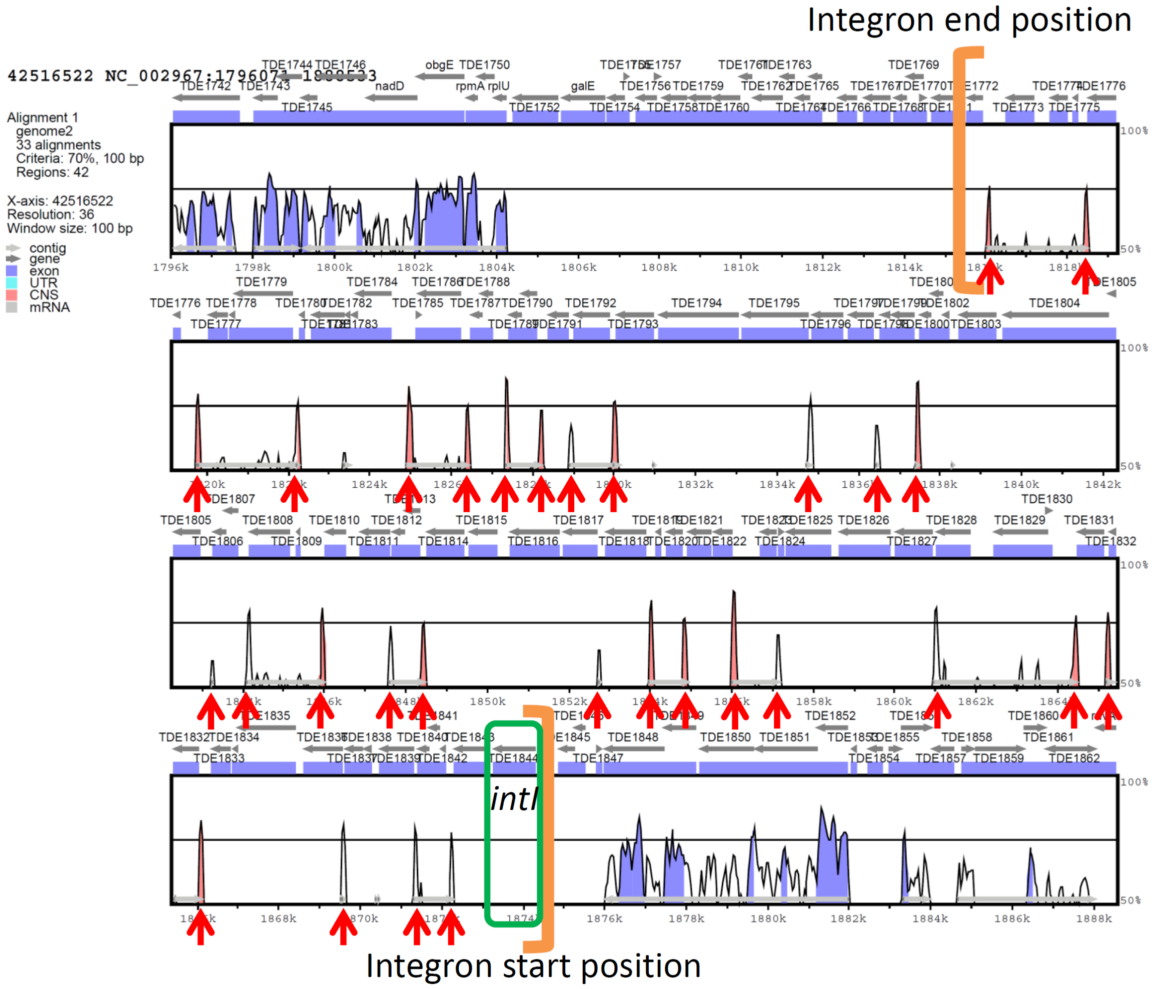

Supplementary Figure 2. Mapping of the *Treponema phagedenis* integron locus to the *Treponema denticola* ATCC 35405 genome. The plot was generated using the wgVista [2] web service, which shows the mapping between the *T. denticola* genome (1,796,071-1,888,533 bp) and all homologous *T. phagedenis* contigs. Regions between these two genomes with sequence similarity are shown as curves in the plots. Higher curves indicate more similarities in the corresponding regions. The integron boundaries are marked by large square brackets, with the location of integron integrase in the *T. denticola* genome highlighted with a green box. The lack of a *T. phagedenis intI* gene can be observed by the lack of conservation (curves) within the green box. The spikes represent *attC* integron recombination sites (noted by red arrowheads) discovered in the *Treponema phagedenis* genome, which are syntenic to the *attC* sites in the *Treponema denticola* genome *(while the gene cassettes between the attC sites are not conserved between these two genomes).* Note that the integron in the *T. denticola* genome is on the reverse-complement strand, so we have marked the start and end positions accordingly.

Supplementary Table 1. Species used in the construction of the phylogenetic tree.

Species	Strain	Accession
Treponema denticola	ATCC 35405	NC_002967
	F0402	Broad institute[1]
	ATCC 35404	Broad institute[1]
	ATCC 33520	Broad institute[1]
	ATCC 33521	Broad institute[1]
	AL-2	Broad institute[1]
	ASLM	Broad institute[1]
	H1-T	Broad institute[1]
	H-22	Broad institute[1]
	MYR-T	Broad institute[1]
	OTK	Broad institute[1]
	SP33	Broad institute[1]
	SP37	Broad institute[1]
	US-Trep	Broad institute[1]
Treponema vincentii	ATCC 35580	HMP reference genome[2]
Treponema phagedenis	F0421	HMP reference genome[2]
Treponema pallidum	SS14	NC_010741
Treponema brennaborense	DSM 12168	NC_015500
Treponema succinifaciens	DSM 2489	NC_015385
Treponema azotonutricium	ZAS-9	NC_015577
Treponema primitia	ZAS-2	NC_015578
Spirochaeta coccoides	DSM 17374	NC_015436
Spirochaete thermophile	DSM 6192	NC_014484
Borrelia burgdorferi	B31	NC_001318
Brachyspira murdochii	DSM 12563	NC_014150

Leptospira interrogans	Lai str. 56601	NC_004342
Xanthomonas campestris	campestris str. 8004	NC_007086
Pseudomonas stutzeri	A1501	NC_009434
Shewanella denitrificans	OS217	NC_007954
Vibrio cholerae	M66-2	NC_012578
Blastopirellula marina	DSM 3645	AANZ00000000
Candidatus Kuenenia		CT030148, CT573074,
Chlorobium	DSM 266	NC_008639
Chlorobium	BS1	NC_010831
Synechococcus	sp.	AANO00000000.1
Salinibacter ruber	DSM 13855	NC_007677

[1]: Treponema denticola F0402 Sequencing Project, Broad Institute of Harvard and MIT (http://www.broadinstitute.org/). [2]: http://hmpdacc.org/.

Supplementary Table 2. Sequences of the eight representative *T. denticola attC* sites.

ID	Sequence
**denti1***	GTCTAACTTCCGCTTAACCTGCATTGCCGCAAAGGCAATGTCAGGTTGAA GCGGGTGTTAGGCAA
**denti2**	GCCTAACATGCACTTAAGGTGCAACTGGGCTGAAGCCCAGTTGTCAGCT TGAAGTGCTTGTTAGAGG
denti3	CGTACAGGCGTTTAACCGGCCGATCTTCAGAGCGGGTTTTGTCAGTCTT GAACTGCGTGTTAGAC
**denti4**	TTCTAACATTCGCCTAATCTGCAAACGGGCATGGTCCCTTTTGTCAGCGT TGAAGTGGGCGTTAGGTA
denti5	CGCAGGCGACCGTCCAAGCAACTTTCGTGACAAAACAAATGTCAGGTTG AAGCGGGTGTTAGACG
denti6	CATATAGGCACATTAAGCAAGTTTTATGACAAAACAAATGTCAGCGTTGG AGCGGGCGTTAGGTT
**denti7**	GTCTAACATTCGCTTAACCTGCATTTGCGGCTTGTCCGCAATGTCAGGTT GAAGCGGGTGTTAGAT
**denti8**	ATCTAACTACCGCTTAACCTGCAACAAGGCTTGTCCTTGTTGTCAGCGTT GAAGCGGGTGTTAGAACT

*: Only five *attC* sites (denti1, denti2, denti4, denti7 and denti8) are included in the alignment shown in Figure 3.

Supplementary Table 3. The locations of integron *attC* recombination sites detected in the draft genomes of 13 *Treponema denticola* strains.

Strain name	Contig id	Start position	End position
al-2	AGDQ01000001	115	175
al-2	AGDQ01000001	2503	2563
al-2	AGDQ01000001	3384	3434
al-2	AGDQ01000001	4476	4528
al-2	AGDQ01000001	5025	5059
al-2	AGDQ01000001	5898	5960
al-2	AGDQ01000004	206226	206279
al-2	AGDQ01000004	293740	293784
al-2	AGDQ01000005	70962	71022
al-2	AGDQ01000006	52061	52124
al-2	AGDQ01000007	201	254
al-2	AGDQ01000007	7464	7526
al-2	AGDQ01000007	8428	8474
al-2	AGDQ01000007	9798	9838
al-2	AGDQ01000007	45013	45067
al-2	AGDQ01000007	46703	46764
al-2	AGDQ01000007	119081	119129
al-2	AGDQ01000015	33	95
al-2	AGDQ01000015	770	820
al-2	AGDQ01000015	1697	1759
al-2	AGDQ01000015	2444	2508
al-2	AGDQ01000015	3561	3623
al-2	AGDQ01000015	4682	4743
al-2	AGDQ01000016	115	169
al-2	AGDQ01000016	2079	2138
al-2	AGDQ01000016	2961	3022
al-2	AGDQ01000017	1	55
al-2	AGDQ01000017	832	885
al-2	AGDQ01000018	110	168
al-2	AGDQ01000018	2349	2412
al-2	AGDQ01000018	5754	5817
al-2	AGDQ01000018	6612	6674
al-2	AGDQ01000018	7989	8050
al-2	AGDQ01000018	8902	8956
al-2	AGDQ01000018	10592	10655
aslm	AGDR01000001	1487	1545
aslm	AGDR01000001	3651	3712

aslm	AGDR01000006	2478	2536
aslm	AGDR01000021	147006	147059
aslm	AGDR01000021	220234	220279
aslm	AGDR01000025	42318	42359
aslm	AGDR01000026	91	152
aslm	AGDR01000026	1181	1243
aslm	AGDR01000026	7875	7936
aslm	AGDR01000026	9180	9242
aslm	AGDR01000026	10951	10986
aslm	AGDR01000031	137	173
aslm	AGDR01000041	18780	18840
aslm	AGDR01000041	19636	19698
aslm	AGDR01000041	20599	20654
aslm	AGDR01000041	20617	20654
aslm	AGDR01000041	21439	21501
aslm	AGDR01000041	22296	22357
aslm	AGDR01000041	24589	24642
aslm	AGDR01000041	25995	26056
aslm	AGDR01000041	26832	26890
aslm	AGDR01000041	27809	27871
aslm	AGDR01000041	31017	31067
aslm	AGDR01000041	31954	32005
aslm	AGDR01000041	32804	32864
aslm	AGDR01000041	33687	33747
aslm	AGDR01000041	34547	34610
aslm	AGDR01000041	35687	35745
aslm	AGDR01000041	36627	36680
aslm	AGDR01000041	39233	39296
aslm	AGDR01000041	40163	40221
aslm	AGDR01000041	43062	43125
aslm	AGDR01000042	478	511
aslm	AGDR01000042	1800	1858
aslm	AGDR01000042	2777	2840
aslm	AGDR01000042	3707	3765
aslm	AGDR01000042	6606	6669
atcc_33520	AGDS01000003	75985	76034
atcc_33520	AGDS01000006	24102	24138
atcc_33520	AGDS01000006	25874	25927
atcc_33520	AGDS01000006	33859	33921
atcc_33520	AGDS01000006	34825	34871
atcc_33520	AGDS01000006	36195	36235
atcc_33520	AGDS01000006	83093	83155
atcc_33520	AGDS01000006	228423	228483

atcc_33520	AGDS01000012	22773	22837
atcc_33520	AGDS01000012	23663	23724
atcc_33520	AGDS01000012	25628	25691
atcc_33520	AGDS01000012	26366	26425
atcc_33520	AGDS01000012	27346	27393
atcc_33520	AGDS01000012	28861	28922
atcc_33520	AGDS01000012	30671	30725
atcc_33520	AGDS01000012	32549	32611
atcc_33520	AGDS01000012	33507	33569
atcc_33520	AGDS01000012	34364	34428
atcc_33520	AGDS01000012	35443	35503
atcc_33520	AGDS01000012	36402	36463
atcc_33520	AGDS01000012	37684	37745
atcc_33520	AGDS01000012	38628	38673
atcc_33520	AGDS01000012	39520	39580
atcc_33520	AGDS01000012	40337	40398
atcc_33520	AGDS01000012	41270	41327
atcc_33520	AGDS01000012	42576	42637
atcc_33520	AGDS01000012	44046	44105
atcc_33520	AGDS01000023	41485	41536
atcc_33520	AGDS01000023	41502	41536
atcc_33520	AGDS01000023	179558	179617
atcc_33520	AGDS01000023	181828	181872
atcc_33521	AGDT01000004	57281	57317
atcc_33521	AGDT01000004	59053	59113
atcc_33521	AGDT01000005	143	207
atcc_33521	AGDT01000005	1678	1743
atcc_33521	AGDT01000005	2500	2561
atcc_33521	AGDT01000005	3857	3918
atcc_33521	AGDT01000005	3857	3893
atcc_33521	AGDT01000005	113500	113548
atcc_33521	AGDT01000005	185418	185478
atcc_33521	AGDT01000005	328413	328448
atcc_33521	AGDT01000014	33	95
atcc_33521	AGDT01000014	1014	1074
atcc_33521	AGDT01000015	31	93
atcc_33521	AGDT01000015	958	1012
atcc_33521	AGDT01000016	32	95
atcc_33521	AGDT01000016	1060	1123
atcc_33521	AGDT01000016	3532	3594
atcc_33521	AGDT01000016	4841	4894
atcc_33521	AGDT01000016	5929	5995
atcc_33521	AGDT01000016	7243	7305

atcc_33521	AGDT01000016	7802	7836
atcc_33521	AGDT01000016	8682	8734
atcc_33521	AGDT01000022	48796	48848
atcc_33521	AGDT01000022	134126	134170
atcc_33521	AGDT01000022	134137	134170
atcc_33521	AGDT01000024	3	65
atcc_33521	AGDT01000024	894	956
atcc_33521	AGDT01000024	1789	1851
atcc_33521	AGDT01000025	839	901
atcc_33521	AGDT01000025	1730	1792
atcc_35404	AGDU01000005	10240	10292
atcc_35404	AGDU01000005	11138	11172
atcc_35404	AGDU01000005	11669	11731
atcc_35404	AGDU01000005	12979	13045
atcc_35404	AGDU01000005	14080	14133
atcc_35404	AGDU01000005	15380	15442
atcc_35404	AGDU01000005	17851	17914
atcc_35404	AGDU01000005	18879	18942
atcc_35404	AGDU01000006	85	149
atcc_35404	AGDU01000006	1014	1076
atcc_35404	AGDU01000007	10	70
atcc_35404	AGDU01000007	989	1051
atcc_35404	AGDU01000018	46541	46576
atcc_35404	AGDU01000019	70363	70423
atcc_35404	AGDU01000020	24038	24086
atcc_35404	AGDU01000020	133668	133729
atcc_35404	AGDU01000020	135025	135086
atcc_35404	AGDU01000020	135843	135908
atcc_35404	AGDU01000020	137379	137443
atcc_35404	AGDU01000021	899	961
atcc_35404	AGDU01000021	7807	7867
atcc_35404	AGDU01000021	9576	9611
atcc_35404	AGDU01000021	9603	9639
atcc_35404	AGDU01000028	146161	146205
atcc_35404	AGDU01000029	32663	32715
f0402	ADEC01000007	119032	119086
f0402	ADEC01000008	315	368
f0402	ADEC01000008	7574	7636
f0402	ADEC01000009	301	342
f0402	ADEC01000011	8983	9038
f0402	ADEC01000011	10010	10043
f0402	ADEC01000011	83039	83094
f0402	ADEC01000011	154397	154457

f0402	ADEC01000014	580903	580964
f0402	ADEC01000014	583062	583125
f0402	ADEC01000014	584026	584081
f0402	ADEC01000014	584867	584932
f0402	ADEC01000014	585848	585895
f0402	ADEC01000014	587050	587104
f0402	ADEC01000014	588410	588458
f0402	ADEC01000014	589854	589918
f0402	ADEC01000014	590582	590644
f0402	ADEC01000014	594428	594491
f0402	ADEC01000014	596873	596932
f0402	ADEC01000014	597489	597548
f0402	ADEC01000014	598293	598345
f0402	ADEC01000014	599131	599192
f0402	ADEC01000014	604072	604133
f0402	ADEC01000014	605106	605168
f0402	ADEC01000014	607073	607131
f0402	ADEC01000014	608253	608312
f0402	ADEC01000014	609318	609379
f0402	ADEC01000014	611291	611349
f0402	ADEC01000014	612186	612248
f0402	ADEC01000014	613053	613108
f0402	ADEC01000014	613848	613909
f0402	ADEC01000014	617020	617081
f0402	ADEC01000016	11042	11086
f0402	ADEC01000019	11902	11954
h1-t	AGDW01000001	211303	211347
h1-t	AGDW01000005	74616	74665
h1-t	AGDW01000009	21155	21191
h1-t	AGDW01000009	22927	22989
h1-t	AGDW01000009	24233	24293
h1-t	AGDW01000009	31069	31130
h1-t	AGDW01000009	32438	32478
h1-t	AGDW01000011	15258	15319
h1-t	AGDW01000011	17200	17262
h1-t	AGDW01000011	94695	94749
h1-t	AGDW01000011	163166	163226
h1-t	AGDW01000016	22141	22204
h1-t	AGDW01000016	23108	23154
h1-t	AGDW01000016	24198	24259
h1-t	AGDW01000017	35	95
h1-t	AGDW01000018	803	862
h1-t	AGDW01000018	1783	1830

h1-t	AGDW01000018	3677	3731
h1-t	AGDW01000018	5555	5617
h1-t	AGDW01000018	6513	6575
h1-t	AGDW01000018	7370	7434
h1-t	AGDW01000018	8449	8509
h1-t	AGDW01000019	1235	1296
h1-t	AGDW01000019	2179	2224
h1-t	AGDW01000019	3071	3131
h1-t	AGDW01000019	3888	3949
h1-t	AGDW01000019	4821	4878
h1-t	AGDW01000019	6127	6188
h1-t	AGDW01000019	7597	7656
h-22	AGDV01000004	49590	49651
h-22	AGDV01000004	50490	50524
h-22	AGDV01000004	51021	51073
h-22	AGDV01000004	52122	52174
h-22	AGDV01000004	53179	53242
h-22	AGDV01000004	54897	54960
h-22	AGDV01000004	58101	58149
h-22	AGDV01000004	59617	59672
h-22	AGDV01000004	61868	61931
h-22	AGDV01000004	62757	62820
h-22	AGDV01000004	63630	63692
h-22	AGDV01000004	65498	65561
h-22	AGDV01000004	66553	66617
h-22	AGDV01000004	67440	67502
h-22	AGDV01000004	68435	68487
h-22	AGDV01000005	591	652
h-22	AGDV01000005	4756	4818
h-22	AGDV01000005	5828	5891
h-22	AGDV01000005	7227	7288
h-22	AGDV01000005	9226	9277
h-22	AGDV01000005	10078	10135
h-22	AGDV01000005	11207	11268
h-22	AGDV01000005	12069	12129
h-22	AGDV01000005	12944	13005
h-22	AGDV01000005	14572	14635
h-22	AGDV01000005	16562	16626
h-22	AGDV01000005	18771	18832
h-22	AGDV01000010	220611	220648
h-22	AGDV01000012	165828	165888
h-22	AGDV01000012	306374	306435
h-22	AGDV01000012	360477	360517

h-22	AGDV01000012	362078	362139
h-22	AGDV01000013	837	900
h-22	AGDV01000014	8125	8165
h-22	AGDV01000020	149928	149972
h-22	AGDV01000020	238595	238647
myr-t	AGDX01000003	21181	21216
myr-t	AGDX01000003	22925	22987
myr-t	AGDX01000003	24231	24291
myr-t	AGDX01000003	31067	31128
myr-t	AGDX01000003	32436	32476
myr-t	AGDX01000003	84065	84126
myr-t	AGDX01000003	86007	86069
myr-t	AGDX01000003	163502	163556
myr-t	AGDX01000003	231973	232033
myr-t	AGDX01000006	606028	606091
myr-t	AGDX01000006	606995	607041
myr-t	AGDX01000006	608085	608146
myr-t	AGDX01000006	609368	609428
myr-t	AGDX01000007	34	95
myr-t	AGDX01000007	1317	1377
myr-t	AGDX01000007	3281	3344
myr-t	AGDX01000008	464	523
myr-t	AGDX01000008	1444	1491
myr-t	AGDX01000008	3338	3392
myr-t	AGDX01000008	5216	5278
myr-t	AGDX01000008	6174	6236
myr-t	AGDX01000008	7031	7095
myr-t	AGDX01000008	8110	8170
myr-t	AGDX01000008	9069	9130
myr-t	AGDX01000008	10352	10413
myr-t	AGDX01000008	11296	11341
myr-t	AGDX01000008	12188	12248
myr-t	AGDX01000008	13005	13066
myr-t	AGDX01000008	13938	13995
myr-t	AGDX01000008	15244	15305
myr-t	AGDX01000008	16714	16773
myr-t	AGDX01000020	28746	28790
myr-t	AGDX01000021	76112	76161
otk	AGDY01000001	199949	200010
otk	AGDY01000001	200862	200921
otk	AGDY01000001	205424	205486
otk	AGDY01000001	210157	210206
otk	AGDY01000001	211739	211792

otk	AGDY01000001	212457	212515
otk	AGDY01000001	214534	214598
otk	AGDY01000001	215424	215489
otk	AGDY01000001	217701	217750
otk	AGDY01000002	13	75
otk	AGDY01000002	1094	1156
otk	AGDY01000002	2722	2767
otk	AGDY01000002	3650	3710
otk	AGDY01000002	5891	5949
otk	AGDY01000002	6868	6929
otk	AGDY01000002	8310	8371
otk	AGDY01000002	9208	9262
otk	AGDY01000002	10238	10303
otk	AGDY01000002	11179	11242
otk	AGDY01000002	12031	12092
otk	AGDY01000002	12892	12943
otk	AGDY01000002	14264	14326
otk	AGDY01000002	14881	14941
otk	AGDY01000002	15681	15742
otk	AGDY01000002	17777	17827
otk	AGDY01000004	321728	321779
otk	AGDY01000005	16593	16628
otk	AGDY01000005	17393	17454
otk	AGDY01000005	20437	20500
otk	AGDY01000006	122202	122250
otk	AGDY01000006	192107	192169
otk	AGDY01000006	192854	192917
otk	AGDY01000006	193781	193845
otk	AGDY01000006	249055	249097
otk	AGDY01000006	249971	250031
otk	AGDY01000006	251728	251790
otk	AGDY01000007	50	113
otk	AGDY01000007	6969	7029
otk	AGDY01000007	7802	7865
otk	AGDY01000009	223272	223324
otk	AGDY01000010	134487	134523
sp33	AGDZ01000005	36527	36579
sp33	AGDZ01000009	15898	15936
sp33	AGDZ01000010	254	303
sp33	AGDZ01000010	1061	1111
sp33	AGDZ01000011	239	293
sp33	AGDZ01000014	51618	51665
sp33	AGDZ01000014	53283	53316

sp33	AGDZ01000018	871	923
sp33	AGDZ01000018	81934	81986
sp33	AGDZ01000018	85363	85423
sp33	AGDZ01000018	86437	86481
sp33	AGDZ01000018	136920	136957
sp33	AGDZ01000018	144973	145009
sp33	AGDZ01000028	91847	91887
sp33	AGDZ01000028	505302	505336
sp33	AGDZ01000029	221	283
sp33	AGDZ01000029	1648	1710
sp33	AGDZ01000029	2511	2569
sp33	AGDZ01000029	4627	4688
sp33	AGDZ01000029	5529	5584
sp33	AGDZ01000030	573	632
sp33	AGDZ01000030	1462	1509
sp33	AGDZ01000031	44	104
sp33	AGDZ01000031	3134	3195
sp33	AGDZ01000032	2	51
sp33	AGDZ01000032	717	780
sp33	AGDZ01000032	3177	3239
sp33	AGDZ01000033	73	135
sp33	AGDZ01000033	1053	1116
sp33	AGDZ01000034	1	35
sp33	AGDZ01000034	1622	1682
sp33	AGDZ01000034	3742	3805
sp33	AGDZ01000034	4846	4910
sp33	AGDZ01000035	1032	1095
sp33	AGDZ01000035	2366	2427
sp33	AGDZ01000036	813	874
sp33	AGDZ01000036	1778	1839
sp33	AGDZ01000036	2560	2615
sp33	AGDZ01000036	3657	3719
sp33	AGDZ01000036	4490	4540
sp33	AGDZ01000036	5285	5349
sp33	AGDZ01000037	1088	1150
sp33	AGDZ01000037	2058	2122
sp33	AGDZ01000037	3061	3116
sp37	AGEA01000002	286010	286061
sp37	AGEA01000004	163647	163682
sp37	AGEA01000004	164447	164508
sp37	AGEA01000004	167723	167785
sp37	AGEA01000005	77430	77483
sp37	AGEA01000006	22018	22070

sp37	AGEA01000006	96113	96175
sp37	AGEA01000006	96860	96923
sp37	AGEA01000006	97788	97851
sp37	AGEA01000006	145389	145430
sp37	AGEA01000006	146305	146365
sp37	AGEA01000006	148062	148122
sp37	AGEA01000006	149504	149565
sp37	AGEA01000007	713	777
sp37	AGEA01000007	1587	1652
sp37	AGEA01000007	2515	2566
sp37	AGEA01000007	3873	3925
sp37	AGEA01000007	10798	10852
sp37	AGEA01000007	11625	11688
sp37	AGEA01000013	171034	171086
sp37	AGEA01000019	50827	50878
sp37	AGEA01000020	1800	1862
sp37	AGEA01000021	4020	4069
sp37	AGEA01000021	5603	5656
sp37	AGEA01000021	6321	6379
sp37	AGEA01000021	8398	8462
sp37	AGEA01000021	9288	9353
sp37	AGEA01000021	11565	11614
sp37	AGEA01000021	12509	12571
sp37	AGEA01000021	13590	13652
sp37	AGEA01000021	15218	15263
sp37	AGEA01000021	16146	16206
sp37	AGEA01000021	18387	18445
sp37	AGEA01000021	18410	18445
sp37	AGEA01000021	19364	19424
sp37	AGEA01000021	20806	20867
sp37	AGEA01000021	21657	21711
sp37	AGEA01000022	1	55
sp37	AGEA01000022	844	905
sp37	AGEA01000022	1705	1756
sp37	AGEA01000022	3077	3139
sp37	AGEA01000023	389	449
sp37	AGEA01000023	1189	1250
sp37	AGEA01000023	3285	3335
us-trep	AGEB01000001	146963	147016
us-trep	AGEB01000001	220191	220236
us-trep	AGEB01000002	65598	65639
us-trep	AGEB01000002	66938	67000
us-trep	AGEB01000002	73633	73694

us-trep	AGEB01000002	74938	75000
us-trep	AGEB01000002	76709	76744
us-trep	AGEB01000004	28701	28737
us-trep	AGEB01000011	32023	32083
us-trep	AGEB01000011	32879	32941
us-trep	AGEB01000011	33842	33897
us-trep	AGEB01000011	34682	34744
us-trep	AGEB01000011	35539	35600
us-trep	AGEB01000011	37832	37885
us-trep	AGEB01000011	39238	39299
us-trep	AGEB01000011	40075	40133
us-trep	AGEB01000011	41052	41114
us-trep	AGEB01000011	44260	44310
us-trep	AGEB01000011	45197	45248
us-trep	AGEB01000011	46047	46107
us-trep	AGEB01000011	46930	46990
us-trep	AGEB01000011	47790	47853
us-trep	AGEB01000011	48930	48988
us-trep	AGEB01000011	49870	49923
us-trep	AGEB01000011	52476	52539
us-trep	AGEB01000012	91	152
us-trep	AGEB01000012	1181	1243
us-trep	AGEB01000012	2259	2292
us-trep	AGEB01000013	186	244
us-trep	AGEB01000013	3085	3148
us-trep	AGEB01000014	310	368
us-trep	AGEB01000014	2474	2535
us-trep	AGEB01000014	2501	2538
us-trep	AGEB01000017	38758	38816

Supplementary Table 4. Integron genes detected in the draft genomes of 13 *Treponema denticola* strains.

Strain name	Contig id	Start position	End position	Sense/Antisense
al-2	AGDQ01000001	2567	2719	+
al-2	AGDQ01000001	2869	3363	+
al-2	AGDQ01000001	3520	3879	+
al-2	AGDQ01000001	3897	4160	+
al-2	AGDQ01000001	4344	4451	+
al-2	AGDQ01000001	5112	5891	+
al-2	AGDQ01000007	7876	8163	+
al-2	AGDQ01000007	8174	8413	+
al-2	AGDQ01000007	8922	9179	+
al-2	AGDQ01000007	45104	45919	+
al-2	AGDQ01000007	45992	46699	+
al-2	AGDQ01000015	856	1686	+
al-2	AGDQ01000015	2989	3378	+
al-2	AGDQ01000015	3733	4608	+
al-2	AGDQ01000016	345	1370	+
al-2	AGDQ01000016	1529	2020	+
al-2	AGDQ01000017	116	430	+
al-2	AGDQ01000017	445	828	+
al-2	AGDQ01000018	295	669	+
al-2	AGDQ01000018	767	1387	+
al-2	AGDQ01000018	1492	2337	+
al-2	AGDQ01000018	2687	4108	+
al-2	AGDQ01000018	4128	5669	+
al-2	AGDQ01000018	7421	7666	+
al-2	AGDQ01000018	7801	7986	+
al-2	AGDQ01000018	8098	8895	+
aslm	AGDR01000001	2278	2709	-
aslm	AGDR01000001	2745	3158	-
aslm	AGDR01000001	3290	3478	-
aslm	AGDR01000026	183	251	-
aslm	AGDR01000026	335	724	-
aslm	AGDR01000026	7949	9127	-
aslm	AGDR01000026	9631	9912	-
aslm	AGDR01000026	9938	10912	-
aslm	AGDR01000041	19080	19301	-
aslm	AGDR01000041	19425	19598	-
aslm	AGDR01000041	19701	20021	-

aslm	AGDR01000041	20660	21079	-
aslm	AGDR01000041	21086	21283	-
aslm	AGDR01000041	22367	23023	-
aslm	AGDR01000041	23110	23655	-
aslm	AGDR01000041	23664	24551	-
aslm	AGDR01000041	26180	26563	-
aslm	AGDR01000041	27878	28816	-
aslm	AGDR01000041	28893	29534	-
aslm	AGDR01000041	29561	30991	-
aslm	AGDR01000041	31068	31910	-
aslm	AGDR01000041	32018	32512	-
aslm	AGDR01000041	35822	36067	-
aslm	AGDR01000041	36155	36508	-
aslm	AGDR01000041	37729	39210	-
aslm	AGDR01000041	39296	39535	-
aslm	AGDR01000041	39540	39857	-
aslm	AGDR01000041	40252	42984	-
aslm	AGDR01000042	666	1607	-
atcc_33520	AGDS01000006	24204	25178	+
atcc_33520	AGDS01000006	25204	25485	+
atcc_33520	AGDS01000006	34273	34560	+
atcc_33520	AGDS01000006	34571	34810	+
atcc_33520	AGDS01000006	35286	35576	+
atcc_33520	AGDS01000012	23894	24919	+
atcc_33520	AGDS01000012	25077	25568	+
atcc_33520	AGDS01000012	26530	26940	+
atcc_33520	AGDS01000012	27190	27309	+
atcc_33520	AGDS01000012	27539	28624	+
atcc_33520	AGDS01000012	28953	30614	+
atcc_33520	AGDS01000012	30746	31417	+
atcc_33520	AGDS01000012	31604	32542	+
atcc_33520	AGDS01000012	33600	34241	+
atcc_33520	AGDS01000012	34538	35404	+
atcc_33520	AGDS01000012	35598	36296	+
atcc_33520	AGDS01000012	36553	36723	+
atcc_33520	AGDS01000012	37031	37129	+
atcc_33520	AGDS01000012	37197	37280	+
atcc_33520	AGDS01000012	38782	39171	+
atcc_33520	AGDS01000012	39335	39415	+
atcc_33520	AGDS01000012	39662	40048	+
atcc_33520	AGDS01000012	40056	40331	+
atcc_33520	AGDS01000012	40426	40617	+
atcc_33520	AGDS01000012	40665	41255	+

atcc_33520	AGDS01000012	41478	42170	+
atcc_33520	AGDS01000012	42179	42265	+
atcc_33520	AGDS01000012	42762	43136	+
atcc_33520	AGDS01000012	43228	43548	+
atcc_33520	AGDS01000012	43938	44039	+
atcc_33520	AGDS01000023	179625	181148	-
atcc_33520	AGDS01000023	181181	181810	-
atcc_33521	AGDT01000004	57383	58357	+
atcc_33521	AGDT01000004	58383	58664	+
atcc_33521	AGDT01000005	338	1558	+
atcc_33521	AGDT01000005	1825	2211	+
atcc_33521	AGDT01000005	2219	2494	+
atcc_33521	AGDT01000005	2742	2831	+
atcc_33521	AGDT01000005	2973	3239	+
atcc_33521	AGDT01000015	121	951	+
atcc_33521	AGDT01000016	125	1054	+
atcc_33521	AGDT01000016	1251	2666	+
atcc_33521	AGDT01000016	2854	3291	+
atcc_33521	AGDT01000016	3307	3471	+
atcc_33521	AGDT01000016	3705	3857	-
atcc_33521	AGDT01000016	3979	4833	+
atcc_33521	AGDT01000016	5358	5747	+
atcc_33521	AGDT01000016	6146	6838	+
atcc_33521	AGDT01000016	6847	6975	+
atcc_33521	AGDT01000016	7889	8668	+
atcc_33521	AGDT01000024	78	230	-
atcc_33521	AGDT01000025	1180	1470	+
atcc_35404	AGDU01000005	10306	11085	-
atcc_35404	AGDU01000005	11999	12127	-
atcc_35404	AGDU01000005	12136	12828	-
atcc_35404	AGDU01000005	13227	13616	-
atcc_35404	AGDU01000005	14141	14995	-
atcc_35404	AGDU01000005	15053	15280	-
atcc_35404	AGDU01000005	15503	15667	-
atcc_35404	AGDU01000005	15683	16120	-
atcc_35404	AGDU01000005	16308	17723	-
atcc_35404	AGDU01000005	17920	18849	-
atcc_35404	AGDU01000006	156	986	-
atcc_35404	AGDU01000020	134347	134613	-
atcc_35404	AGDU01000020	134755	134844	-
atcc_35404	AGDU01000020	135092	135367	-
atcc_35404	AGDU01000020	135375	135761	-
atcc_35404	AGDU01000020	136028	137248	-

atcc_35404	AGDU01000021	8256	8537	-
atcc_35404	AGDU01000021	8563	9537	-
f0402	ADEC01000014	583128	583490	-
f0402	ADEC01000014	584087	584506	-
f0402	ADEC01000014	584513	584710	-
f0402	ADEC01000014	584932	585171	-
f0402	ADEC01000014	585176	585508	-
f0402	ADEC01000014	587172	588017	-
f0402	ADEC01000014	588094	588201	-
f0402	ADEC01000014	588567	589814	-
f0402	ADEC01000014	594570	595826	-
f0402	ADEC01000014	597039	597413	-
f0402	ADEC01000014	597896	598285	-
f0402	ADEC01000014	598348	598806	-
f0402	ADEC01000014	604410	605066	-
f0402	ADEC01000014	605228	605719	-
f0402	ADEC01000014	605878	606903	-
f0402	ADEC01000014	607138	607557	-
f0402	ADEC01000014	607565	607762	-
f0402	ADEC01000014	607787	608212	-
f0402	ADEC01000014	609417	609806	-
f0402	ADEC01000014	610040	611224	-
f0402	ADEC01000014	611362	611601	-
f0402	ADEC01000014	611612	611899	-
f0402	ADEC01000014	612259	612753	-
h1-t	AGDW01000009	21257	22231	+
h1-t	AGDW01000009	22257	22538	+
h1-t	AGDW01000009	23042	24220	+
h1-t	AGDW01000009	31529	31819	+
h1-t	AGDW01000011	16769	17017	+
h1-t	AGDW01000011	17054	17140	+
h1-t	AGDW01000016	22556	22843	+
h1-t	AGDW01000016	22854	23093	+
h1-t	AGDW01000016	23311	23445	+
h1-t	AGDW01000016	23626	24015	+
h1-t	AGDW01000018	967	1377	+
h1-t	AGDW01000018	1884	3620	+
h1-t	AGDW01000018	3800	4423	+
h1-t	AGDW01000018	4610	5548	+
h1-t	AGDW01000018	6588	7247	+
h1-t	AGDW01000018	7544	8410	+
h1-t	AGDW01000019	2333	2722	+
h1-t	AGDW01000019	3213	3599	+

h1-t	AGDW01000019	3607	3882	+
h1-t	AGDW01000019	3977	4168	+
h1-t	AGDW01000019	4216	4806	+
h1-t	AGDW01000019	5029	5721	+
h1-t	AGDW01000019	6313	6687	+
h1-t	AGDW01000019	6779	7099	+
h1-t	AGDW01000019	7489	7590	+
h-22	AGDV01000004	49658	50437	-
h-22	AGDV01000004	51389	51652	-
h-22	AGDV01000004	51670	52029	-
h-22	AGDV01000004	52188	52445	-
h-22	AGDV01000004	52487	52696	-
h-22	AGDV01000004	54346	54861	-
h-22	AGDV01000004	58258	59505	-
h-22	AGDV01000004	59682	61838	-
h-22	AGDV01000004	61931	62170	-
h-22	AGDV01000004	62175	62507	-
h-22	AGDV01000004	62829	63578	-
h-22	AGDV01000004	63699	64490	-
h-22	AGDV01000004	64578	64895	-
h-22	AGDV01000004	65065	65457	-
h-22	AGDV01000004	65563	66495	-
h-22	AGDV01000004	66630	66869	-
h-22	AGDV01000004	66880	67167	-
h-22	AGDV01000004	67512	67676	-
h-22	AGDV01000004	67780	68136	-
h-22	AGDV01000005	662	1492	-
h-22	AGDV01000005	1578	3221	-
h-22	AGDV01000005	3386	4756	-
h-22	AGDV01000005	5895	6740	-
h-22	AGDV01000005	6745	7170	-
h-22	AGDV01000005	7436	8137	-
h-22	AGDV01000005	8243	9196	-
h-22	AGDV01000005	9282	9956	-
h-22	AGDV01000005	10186	10461	-
h-22	AGDV01000005	10482	11171	-
h-22	AGDV01000005	12712	12906	-
h-22	AGDV01000005	13010	13969	-
h-22	AGDV01000005	14140	14523	-
h-22	AGDV01000005	14695	15186	-
h-22	AGDV01000005	15344	16369	-
h-22	AGDV01000005	16732	18012	-
h-22	AGDV01000005	18314	18454	-

h-22	AGDV01000012	361135	361425	-
h-22	AGDV01000012	361799	361987	-
myr-t	AGDX01000003	21255	22229	+
myr-t	AGDX01000003	22255	22536	+
myr-t	AGDX01000003	23040	24218	+
myr-t	AGDX01000003	31527	31817	+
myr-t	AGDX01000003	85576	85824	+
myr-t	AGDX01000003	85861	85947	+
myr-t	AGDX01000006	606443	606730	+
myr-t	AGDX01000006	606741	606980	+
myr-t	AGDX01000006	607198	607332	+
myr-t	AGDX01000006	607513	607902	+
myr-t	AGDX01000006	608236	608469	+
myr-t	AGDX01000006	608794	608964	+
myr-t	AGDX01000007	1547	2572	+
myr-t	AGDX01000007	2730	3221	+
myr-t	AGDX01000008	628	1038	+
myr-t	AGDX01000008	1288	1407	+
myr-t	AGDX01000008	1545	3281	+
myr-t	AGDX01000008	3413	4084	+
myr-t	AGDX01000008	4271	5209	+
myr-t	AGDX01000008	6267	6908	+
myr-t	AGDX01000008	7205	8071	+
myr-t	AGDX01000008	8265	8963	+
myr-t	AGDX01000008	9699	9797	+
myr-t	AGDX01000008	11450	11839	+
myr-t	AGDX01000008	12003	12083	+
myr-t	AGDX01000008	12330	12716	+
myr-t	AGDX01000008	12724	12999	+
myr-t	AGDX01000008	13094	13285	+
myr-t	AGDX01000008	13333	13923	+
myr-t	AGDX01000008	14146	14838	+
myr-t	AGDX01000008	14847	14933	+
myr-t	AGDX01000008	15430	15804	+
myr-t	AGDX01000008	15896	16216	+
myr-t	AGDX01000008	16606	16707	+
otk	AGDY01000001	210293	210703	-
otk	AGDY01000001	210906	211697	-
otk	AGDY01000001	213248	214129	-
otk	AGDY01000001	214261	214449	-
otk	AGDY01000001	214598	214837	-
otk	AGDY01000001	214842	215174	-
otk	AGDY01000001	215493	217688	-

otk	AGDY01000002	135	221	-
otk	AGDY01000002	260	649	-
otk	AGDY01000002	818	952	-
otk	AGDY01000002	3724	5436	-
otk	AGDY01000002	5525	5761	-
otk	AGDY01000002	7126	7401	-
otk	AGDY01000002	7653	8255	-
otk	AGDY01000002	8990	9163	-
otk	AGDY01000002	9293	9565	-
otk	AGDY01000002	9664	9879	-
otk	AGDY01000002	10374	10625	-
otk	AGDY01000002	10711	11067	-
otk	AGDY01000002	11248	11667	-
otk	AGDY01000002	11675	11875	-
otk	AGDY01000002	12105	12599	-
otk	AGDY01000002	12950	13522	-
otk	AGDY01000002	13572	13862	-
otk	AGDY01000002	14433	14843	-
otk	AGDY01000002	14945	15661	-
otk	AGDY01000002	15742	16452	-
otk	AGDY01000002	16545	17735	-
otk	AGDY01000005	16815	17147	+
otk	AGDY01000006	192180	192815	-
otk	AGDY01000006	192924	193754	-
otk	AGDY01000006	249209	249292	-
otk	AGDY01000006	249360	249632	-
otk	AGDY01000007	7418	7699	-
sp33	AGDZ01000010	552	854	+
sp33	AGDZ01000010	931	1053	+
sp33	AGDZ01000018	85429	85767	-
sp33	AGDZ01000018	85982	86143	-
sp33	AGDZ01000029	291	1610	-
sp33	AGDZ01000029	2574	3176	-
sp33	AGDZ01000029	3262	3612	-
sp33	AGDZ01000029	3704	4594	-
sp33	AGDZ01000029	4836	5078	-
sp33	AGDZ01000029	5189	5491	-
sp33	AGDZ01000032	922	2886	-
sp33	AGDZ01000034	39	746	-
sp33	AGDZ01000034	1167	1622	-
sp33	AGDZ01000034	2368	3708	-
sp33	AGDZ01000034	3879	4712	-
sp33	AGDZ01000035	1098	1418	-

sp33	AGDZ01000035	1760	2329	-
sp33	AGDZ01000036	888	1040	-
sp33	AGDZ01000036	1142	1420	-
sp33	AGDZ01000036	1844	2518	-
sp33	AGDZ01000036	3728	4444	-
sp33	AGDZ01000036	4548	5078	-
sp33	AGDZ01000037	1252	1950	-
sp33	AGDZ01000037	2129	2926	-
sp37	AGEA01000004	163869	164201	+
sp37	AGEA01000004	164206	164445	+
sp37	AGEA01000006	96186	96821	-
sp37	AGEA01000006	96930	97760	-
sp37	AGEA01000006	145543	145626	-
sp37	AGEA01000006	145694	145966	-
sp37	AGEA01000006	148320	148595	-
sp37	AGEA01000006	148847	149449	-
sp37	AGEA01000007	786	1535	-
sp37	AGEA01000007	1659	2471	-
sp37	AGEA01000007	2575	2850	-
sp37	AGEA01000007	2980	3201	-
sp37	AGEA01000007	3412	3687	-
sp37	AGEA01000007	11241	11480	-
sp37	AGEA01000021	4156	4440	-
sp37	AGEA01000021	4770	5561	-
sp37	AGEA01000021	7112	7993	-
sp37	AGEA01000021	8125	8313	-
sp37	AGEA01000021	8706	9023	-
sp37	AGEA01000021	9357	11552	-
sp37	AGEA01000021	11619	11981	-
sp37	AGEA01000021	12756	13145	-
sp37	AGEA01000021	13314	13448	-
sp37	AGEA01000021	16220	17932	-
sp37	AGEA01000021	18021	18257	-
sp37	AGEA01000021	20884	21279	-
sp37	AGEA01000021	21439	21612	-
sp37	AGEA01000021	21742	22014	-
sp37	AGEA01000021	22113	22328	-
sp37	AGEA01000022	61	480	-
sp37	AGEA01000022	488	688	-
sp37	AGEA01000022	918	1412	-
sp37	AGEA01000022	1763	2317	-
sp37	AGEA01000022	2620	2895	-
sp37	AGEA01000023	453	1169	-

sp37	AGEA01000023	1250	1960	-
sp37	AGEA01000023	2053	3243	-
us-trep	AGEB01000002	66257	66547	-
us-trep	AGEB01000002	66705	66854	+
us-trep	AGEB01000002	73707	74885	-
us-trep	AGEB01000002	75389	75670	-
us-trep	AGEB01000002	75696	76670	-
us-trep	AGEB01000011	32177	32302	-
us-trep	AGEB01000011	32323	32544	-
us-trep	AGEB01000011	32668	32841	-
us-trep	AGEB01000011	32944	33132	-
us-trep	AGEB01000011	33264	33509	-
us-trep	AGEB01000011	33903	34322	-
us-trep	AGEB01000011	34329	34526	-
us-trep	AGEB01000011	35610	36266	-
us-trep	AGEB01000011	36353	36898	-
us-trep	AGEB01000011	36907	37794	-
us-trep	AGEB01000011	39423	39806	-
us-trep	AGEB01000011	41121	42059	-
us-trep	AGEB01000011	42136	42777	-
us-trep	AGEB01000011	42804	44234	-
us-trep	AGEB01000011	44311	45153	-
us-trep	AGEB01000011	45261	45755	-
us-trep	AGEB01000011	49065	49310	-
us-trep	AGEB01000011	49398	49751	-
us-trep	AGEB01000011	50972	52453	-
us-trep	AGEB01000012	183	251	-
us-trep	AGEB01000012	335	724	-
us-trep	AGEB01000012	1257	1409	-
us-trep	AGEB01000012	1511	1789	-
us-trep	AGEB01000013	275	3007	-
us-trep	AGEB01000014	1101	1532	-
us-trep	AGEB01000014	1568	1981	-
us-trep	AGEB01000014	2113	2301	-

Supplementary Table 5. Summary of the integron genes detected in the draft genomes of 13 *Treponema denticola* strains.

Strains	Number of contigs carrying integron cassette genes	Total cassette genes
al-2	9	26
aslm	4	29
atcc_33520	4	31
atcc_33521	6	20
atcc_35404	4	18
f0402	1	23
h1-t	5	25
h-22	3	38
myr-t	4	34
otk	5	34
sp33	8	24
sp37	6	37
us-trep	5	32

References

1. Cambray G, Guerout AM, Mazel D: **Integrons**. *Annu Rev Genet* 2010, **44**:141-166.
2. Couronne O, Poliakov A, Bray N, Ishkhanov T, Ryaboy D, Rubin E, Pachter L, Dubchak I: **Strategies and tools for whole-genome alignments**. *Genome Res* 2003, **13**(1):73-80.
